# Nutritionally Important Pro-Health Active Ingredients and Antioxidant Properties of Fruits and Fruit Juice of Selected Biennial Fruiting *Rubus idaeus* L. Cultivars

**DOI:** 10.3390/ph16121698

**Published:** 2023-12-07

**Authors:** Mirosława Chwil, Renata Matraszek-Gawron, Mikołaj Kostryco, Monika Różańska-Boczula

**Affiliations:** 1 Department of Botany and Plant Physiology, University of Life Sciences in Lublin, Akademicka 15, 20-950 Lublin, Poland; mikolaj.kostryco@up.lublin.pl; 2 Department of Applied Mathematics and Computer Science, University of Life Sciences in Lublin, Akademicka 13, 20-950 Lublin, Poland; monika.boczula@up.lublin.pl

**Keywords:** raspberry, *Rubi idaei fructus*, bioactive compounds, fatty acids, amino acid, flavonoids

## Abstract

Raspberry fruits are an important source of many biologically active chemical compounds exerting nutritional and pro-health effects. The study presents a comparative analysis of nutritionally important bioactive chemical compounds—polyphenols; flavonoids, including anthocyanins; vitamin C; amino acids; fatty acids; and primary metabolites—contained in the fruits of three biennial fruiting cultivars, *R. idaeus* ‘Glen Ample’, ‘Laszka’, and ‘Radziejowa’, i.e., common cultivars in Poland and Europe. The antioxidant activity of fresh fruits and juice was determined with five methods. The analyses revealed the strong free radical scavenging potential of the fruits and juice, confirmed by the high concentration of nutrients, e.g., polyphenols, anthocyanins, vitamin C, amino acids, and fatty acids. The antioxidant activity of the juice determined with the ferric reducing antioxidant power (FRAP) and OH radical methods was from 2.5 to 4.0 times higher than that of the fruits. The following orders of total polyphenol contents were established in the analyzed cultivars: ‘Glen Ample’ < ‘Laszka’ < ‘Radziejowa’ in the fruits and ‘Glen Ample’ < ‘Radziejowa’ < ‘Laszka’ in the juice. The highest antioxidant activity was exhibited by the ‘Radziejowa’ fruits. Given their high content of dietary fiber, the fruits of the analyzed raspberry cultivars can be consumed by dieting subjects. The concentrations of vitamin C (28–34 mg/100 g) and anthocyanins (20–34 mg/100 g) indicate the biological and pharmacological activity of these fruits. The main unsaturated fatty acids in the fruits were gamma-linoleic acid (C18:2n6c) and alpha-linolenic acid (C18:3n3), which neutralize excess free radicals. The amino acids nutritionally essential to humans were dominated by leucine, arginine, and phenylalanine. This is the first comparative analysis of the antioxidant activity of fruits and juice and the contents of selected active compounds in the fruits of biennial fruiting cultivars of *R. idaeus*, i.e., a highly commercialized crop in Europe.

## 1. Introduction

Given the increased incidence of lifestyle diseases, scientists are currently searching for natural bioactive compounds with a broad spectrum of activity but no side effects to be used in the production of pharmaceutical and cosmetic formulations.

### 1.1. Oxidative Stress and Its Effects and Defense Mechanisms against ROS

Oxidative stress plays an important role in the etiology and course of lifestyle diseases. It is associated with a disturbed balance between oxidants and antioxidants having an ability to detoxify the highly reactive by-products of metabolic transformations [1,2]. Reactive oxygen (ROS) and reactive nitrogen (RNS) species include oxygen free radicals and non-radical derivatives [3,4]. The most important sites of ROS generation in a plant cell include the chloroplasts, peroxisomes, and mitochondria. Due to the large amounts of ROS formed in the chloroplasts, proteins involved in photosynthetic electron transport are often oxidized [4,5]. At moderate concentrations, ROS and RNS mediate the regulation of cell signaling transduction processes, and their excessive production activates nucleases, damages (deoxyribonucleic acid) DNA, and results in cytotoxic activity [6,7,8]. Oxidative stress damages lipids and oxidizes cell membranes, changes the structure and modifies the functions of proteins due to the oxidation of amino acid residues, and damages carbohydrates by breaking the glycosidic bonds of polysaccharides [9,10]. It reduces adenosine triphosphate (ATP) production via the inhibition of oxidative phosphorylation, leading to the oxidation of low-molecular compounds [11,12], and induces the formation of free radical oxidation products, resulting in apoptotic cell death [13,14].

The enzymatic system protecting cells from ROS includes superoxide dismutase, which catalyzes O_2_^•−^ dismutation, and catalase participating in H_2_O_2_ disproportionation [15]. Enzymes associated with the glutathione–ascorbic pathway, i.e., ascorbate peroxidase, dehydroascorbate reductase, and glutathione reductase, are involved in ROS detoxification as well [16,17]. In the non-enzymatic system, the small-molecular antioxidant compounds acting as scavengers are represented by membrane-active lipophilic carotenoids, flavonoids, and vitamins C and B_6_ [18,19]. Vitamin C scavenges ^1^O_2_, OH^•^, O_2_^•−^ superoxide radicals, and ONOOH; acts a cofactor for ROS detoxifying enzymes; and regulates cell metabolism systems [17,20].

### 1.2. Rubi idaei fructus as a Source of Antioxidants

Recently, interest in berries has significantly increased together with the increased consumption associated with their high nutritional value and phytotherapeutic properties. *Rubi idaei fructus* is a promising source of antioxidants that can serve as substitutes for synthetic agents in prophylaxis and adjuvant therapies [21,22]. *R. idaeus* is an important species for consumption, processing, medicinal, and cosmetic purposes. The majority of commercial cultivars derive from red raspberry [23]. On a global scale, the cultivation area of this species is expanding [24,25,26]. New fruit storage technologies contribute to the maintenance of an unchanged chemical profile, i.e., the content of anthocyanins, vitamins, and tannins. These phytochemicals exhibit antioxidant [27], anti-inflammatory [28], antibacterial [29], immunomodulatory [30], and anticancer activity [31].

### 1.3. Use of R. idaeus Fruits

Due to their rich chemical composition and pro-health (e.g., antioxidant) activity, *R. idaeus* fruits are an important component of diets [32], functional foods [33], nutraceuticals [34], supplements [35], medicines [36], and natural cosmetics [37]. The bioactive compounds contained in raspberry fruit exert a wide range of beneficial phytotherapeutic effects [38]. The fruits are recommended as part of the prophylaxis of metabolic diseases, e.g., cardiovascular diseases [24], diabetes [39], obesity [40], and Alzheimer’s disease [41]. The anti-inflammatory activity of *Rubi idaei fructus* is associated with the inhibitory effect of its compounds on lipoxygenase and cyclooxygenase-2 activity [28]. Additionally, extracts from red raspberries can be used in the prophylaxis and treatment of *Helicobacter pylori* infection [42]. A combination of prebiotics and active chemical compounds from *Rubi idaei fructus* is a natural antimicrobial agent to be used in the production of functional foods and in other branches of the food industry [43]. Raspberry fruit extracts may serve as chemotherapeutic agents, as they have been found to inhibit the migration and invasion of nasopharyngeal cancer cells, the expression of MMP-2, and the ERK1/2 signaling pathway [44]. *Rubi idaei *fructus** fruits are characterized by high antioxidant activity [45,46,47]. These raw materials contain enzymatic and non-enzymatic antioxidants inducing the action of enzymes [28,46,48].

Biologically active phenolic compounds are mainly regarded as the most abundant antioxidants in the diet [49]. The total content of phenolic compounds determined with the Folin–Ciocalteau method confirms that raspberry fruits are a good source of antioxidant secondary metabolites [46,50,51]. The methods used, i.e., the ferric reducing antioxidant power (FRAP), 2,2-diphenyl-1-picrylhydrazyl (DPPH) radical, 2,2’-azinobis-(3-ethyl-benzothiazoline-6-sulfonic acid) (ABTS) radical, and OH^•^ scavenging assays, confirm the high free radical scavenging activity of secondary metabolites from *Rubi idaei fructus* raw material [28,29,47,50,51,52,53,54,55]. This indicates that raspberry fruits can be used as a valuable nutritional component in the adjuvant therapy of oxidative stress-related diseases.

### 1.4. Selected Methods for the Determination of Oxidative Activity

The comparative analysis of antioxidant properties is based on several oxidation–reduction reactions taking place between an oxidant (a reagent appropriate for a given method, e.g., ABTS cation radical, DPPH, OH radical, FRAP reagent, and Folin) and a reducer, i.e., the sum of antioxidant compounds present in the raw material, e.g., *Rubus idaei fructus*. The content of antioxidants present in a sample is determined based on the decrease or increase in the absorbance of the reaction mixture measured spectrophotometrically at a specific wavelength. The Folin–Ciocalteau procedure is based on the use of Folin’s reagent, i.e., a mixture of sodium tungstate (Na_2_WO_4_), sodium molybdate (Na_2_MoO_4_), lithium sulphate (Li_2_SO_4_), bromine water, and concentrated hydrochloric acid and phosphoric acid [56]. In the ABTS and DPPH methods, 2,2’-azinobis(3ethylbenzothiazoline-6-sulfonate) and 2,2-diphenyl-1-picrohydrazyl reagents, respectively, react with antioxidants contained in the raw material and cause discoloration of cation radicals. The degree of discoloration is proportional to the content of the antioxidant compound and is monitored spectrophotometrically at 734 nm and 515 nm wavelengths [57,58]. The FRAP method involves the reaction of the iron-2,4,6-tripyridyl-S-triazine complex obtained from the reaction of 2,4,6-tripyridyl-S-triazine (TPTZ) with iron chloride (FeCl_3_) in acetic buffer (pH 3.5) with antioxidants present in the raw material. The complex becomes intensely blue, and its concentration is monitored using a spectrophotometer at a wavelength of 593 nm. The principle of the OH^•^ radical-based method is to produce the radical in the Fenton reaction in a medium with iron sulfate, hydrogen peroxide, and sodium salicylate, measured at a wavelength of 562 nm [59]. These methods facilitate a comparative analysis of the antioxidant capacity of selected raw materials, e.g., *R. idaeus* fruits and juice.

Although the chemical composition of fruits of many *Rubus* species has widely been investigated, the literature does not provide comparative analyses of the antioxidant activities, fatty acid contents, and energy values of popular raspberry cultivars grown commercially in Poland and Europe. These parameters are important, as fresh raspberry fruits can be consumed for a long time during the season, and processed raspberries are a component of dietary, pharmaceutical, and nutraceutical products throughout the year. The biennial fruiting *R. idaeus* cultivars ‘Glen Ample’, ‘Laszka’, and ‘Radziejowa’ are grown in commercial plantations in Poland and many parts of Europe. In Poland, these cultivars have been listed in the National Register of Horticultural Plant Varieties. Given the popularity of these *R. idaeus* cultivars and the lack of comparative data on the bioactive substances present in these cultivars in the literature, this study is an attempt to provide the missing data and represents a further stage in the investigation of this raw material. The aim of the study was to compare (i) the antioxidant activities in raspberry fruits and fruit juice and other parameters only in the fruits of three *R. idaeus* cultivars, ‘Glen Ample’, ‘Laszka’, and ‘Radziejowa’: (ii) the energy values and contents of (iii) available carbohydrates, (iv) total sugars, (v) total protein, (vi) total fats, (vii) anthocyanins, (viii) total fiber, (ix) ash, and (x) water, as well as the qualitative and quantitative composition of (xi) amino acids and (xii) fatty acids.

## 2. Results

### 2.1. Antioxidant Activity and Content of Polyphenols in Raspberry Fruits and Juice

The FRAP method showed the highest values of antioxidant activity in the ‘Radziejowa’ fruits (9.76 μM Fe(II)/g) and the lowest level in the ‘Laszka’ cultivar (8.28 μmol Fe(II)/g). In the analysis of the fruit juice, the highest reducing power was found for ‘Laszka’ (29.06 μmol Fe(II)/mL), followed by ‘Radziejowa’ (25.67 μmol Fe(II)/mL) and ‘Glen Ample’ (23.66 μmol Fe(II)/mL).

In the DPPH method, the ‘Radziejowa’ fruits exhibited the highest antioxidant capacity with the highest AE coefficient (1.32 mg TE/g), and the lowest value was calculated in the case of ‘Glen Ample’ (1.07 mg TE/g). This parameter in the fresh fruit juice ranged between 2.44 mg TE/g (‘Radziejowa’) and 3.01 mg TE/g (‘Laszka’).

In the ABTS method, the antioxidant activity of the fresh fruit and juice was in the range from 2.31 in ‘Glen Ample’ to 3.17 mg TE/g in ‘Radziejowa’ and from 3.16 in ‘Radziejowa’ to 3.62 mg TE/mL in ‘Laszka’, respectively. The antioxidant activity of the fruits of the three cultivars (mg TE/g) assessed by the OH^•^ scavenging assay was 17.93 in ‘Laszka’, 19.34 in ‘Radziejowa’, and 19.47 in ‘Glen Ample’, while the antioxidant activity results for the juice (mg TE/mL) were as follows: 72.32 in ‘Radziejowa’, 75.49 in ‘Laszka’, and 76.77 in ‘Glen Ample’.

The total polyphenol content in the fresh fruits of the three raspberry cultivars determined with the Folin–Ciocalteu method ranged from 0.99 (‘Glen Ample’) to 1.31 mg/g (caffeic acid equivalents) (‘Radziejowa’). In turn, the concentration of polyphenols in the raspberry juice was between 1.26 (‘GlenAmple’) and 1.79 mg/mL (‘Laszka’). These values differed significantly between the cultivars (Table 1 and Table 2).

### 2.2. Energy Value of Fruits

**Figure 1:**
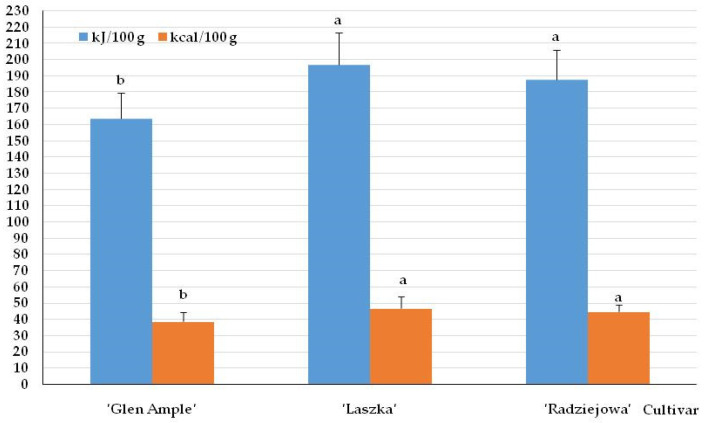
Energy values of the fresh fruits of the biennial fruiting *Rubus idaeus* cultivars. Notes: Means of each trait followed by the same letter do not differ between the cultivars at a significance level of α = 0.05. Vertical bars represent the standard deviations of the means.

The energy value of the fruits of the analyzed *R. idaeus* cultivars ranged from 164 in ‘Glen Ample’ to 197 kJ/100 g in ‘Laszka’. This parameter converted into kcal/100 g was in the range of 39–47. The value of this parameter in ‘Glen Ample’ was significantly lower than in ‘Laszka’ and ‘Radziejowa’ (Figure 1).

### 2.3. Total Content of Sugars, Carbohydrates, Fiber, Protein, and Lipids in Raspberry Fruits

**Figure 2:**
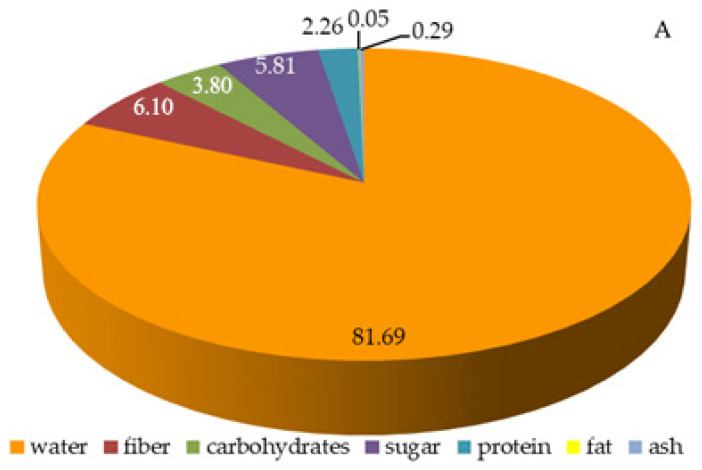

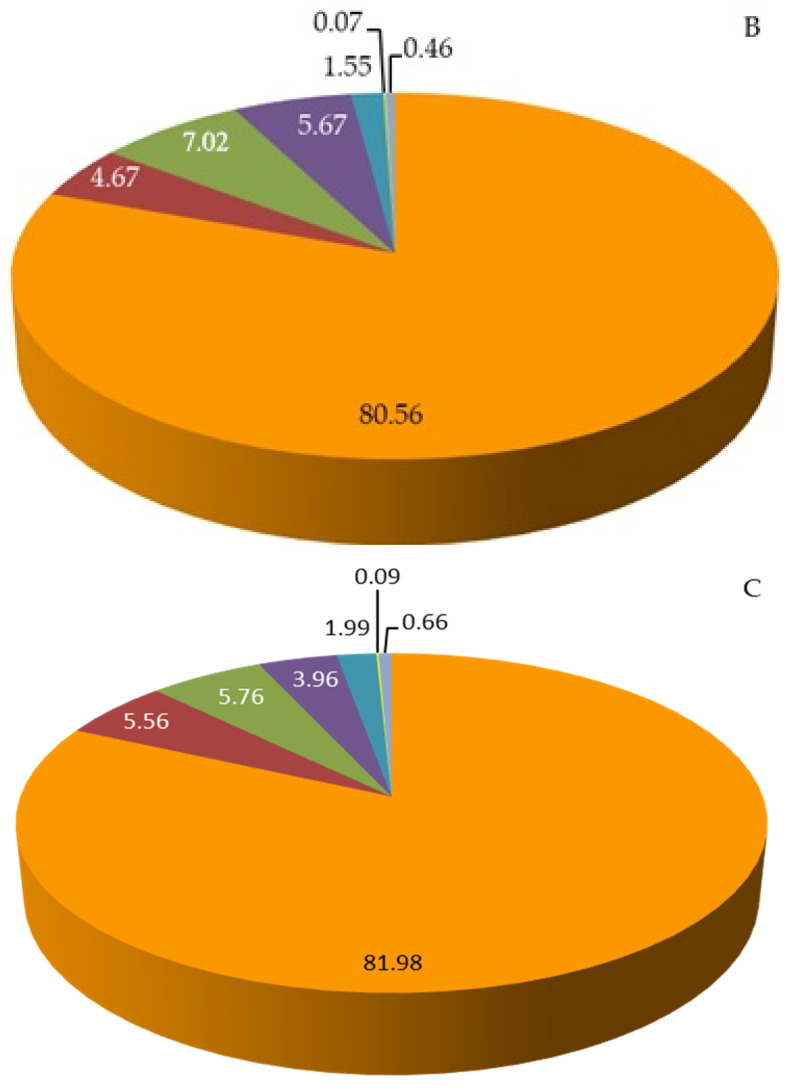
(**A**–**C**) Percentage contents of water, available carbohydrates, total sugars, total fiber, protein, fat, and ash in the fruits of the biennial fruiting *R. idaeus* cultivars: (**A**) ‘Glen Ample’, (**B**) ‘Laszka’, (**C**) ‘Radziejowa’.

The total protein content in the fruits of the analyzed raspberry cultivars ranged between 1.55 in ‘Laszka’ and 2.26 g/100 g in ‘Glen Ample’. The total fat content in the three cultivars was approximately 0.1 g/100 g. The total sugar content in the analyzed fruits ranged from 3.96 (‘Radziejowa’) to 5.81 g/100 g ‘Glen Ample’. The concentration of carbohydrates ranged from 3.80 (‘Glen Ample’) to 7.02 g/100 g (‘Laszka’). The total fiber content was between 4.67 (‘Laszka’) and 6.10 g/100 (‘Glen Ample’). The water content ranged from 80.56 (‘Laszka’) to 8681.98 (‘Radziejowa’) g/100 (Figure 2A–C).

### 2.4. Flavonoids

**Figure 3:**
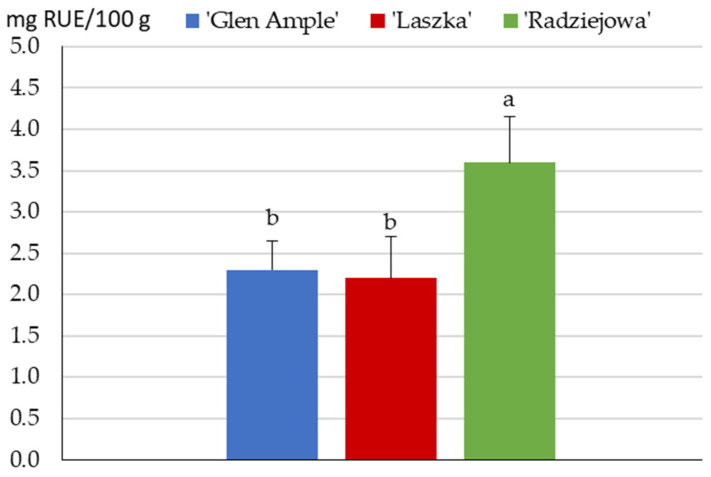
Total contents of flavonoids (mg RUE/g) in the fruits of the biennial fruiting *R. idaeus* cultivars. Notes: Means for total flavonoid content followed by the same letter do not differ between the cultivars at a significance level of α = 0.05. Vertical bars represent the standard deviations of the means.

The content of flavonoids in the fruits of the three raspberry cultivars ranged from 2.2 in ‘Laszka’ to 3.6 mg RUE/100 g in ‘Radziejowa’ (calculated as rutin). The contents of flavonoids in ‘Glen Ample’ and ‘Laszka’ were 36% and 39% lower, respectively, than in ‘Radziejowa’. The content of flavonoids in the ‘Radziejowa’ fruits was markedly higher than in the ‘Glen Ample’ and ‘Laszka’ cultivars (Figure 3).

### 2.5. Content of Anthocyanins and Vitamin C

**Figure 4:**
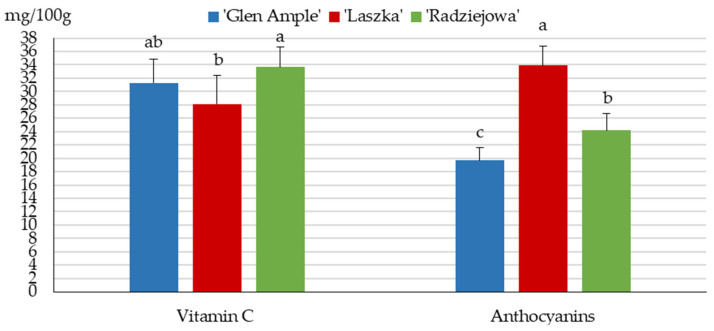
Total contents of vitamin C and anthocyanins (mg/100 g f.w.) in the fruits of the biennial fruiting *R. idaeus* cultivars. Notes: Means for vitamin C and anthocyanin content followed by the same letter do not differ between the cultivars at a significance level of α = 0.05. Vertical bars represent the standard deviations of the means.

Anthocyanins are another important group of bioactive chemical compounds in terms of antioxidant properties. The total content of anthocyanins in the fruits of the analyzed *R. idaeus* cultivars ranged between 19.7 in ‘Glen Ample’ and 33.9 mg/100 g f.w. in ‘Laszka’. The value of this parameter in ‘Radziejowa’ was significantly higher than in ‘Glen Ample’ and markedly lower than in ‘Laszka’. The content of vitamin C in the fruits ranged from 28.15 in ‘Laszka’ to 33.64 mg/100 g f.w. in ‘Radziejowa’, and the value of this parameter in ‘Laszka’ was comparable to that for ‘Glen Ample’ and markedly lower than in ‘Radziejowa’ (Figure 4).

### 2.6. Amino Acids

**Figure 5:**
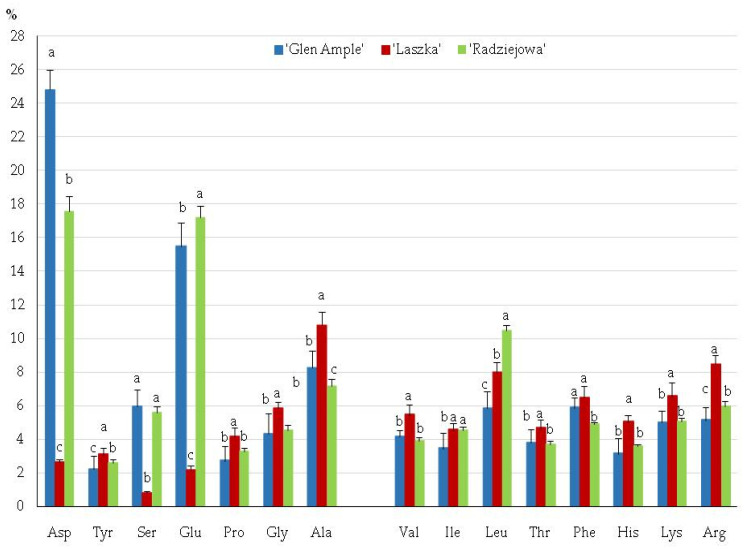
Amino acid contents in the fruits of the biennial fruiting *R. idaeus* cultivars. Notes: Means of each amino acid followed by the same letter do not differ between the cultivars at a significance level of α = 0.05. Vertical bars represent the standard deviations of the means.

In the fruits of the examined *R. idaeus* cultivars, ‘Glen Ample’, ‘Laszka’, and ‘Radziejowa’, the presence of 15 protein amino acids was determined. The total sums of protein amino acids in the *Rubi idaei fructus* of ‘Glen Ample’, ‘Laszka’, and ‘Radziejowa’ were 9.11, 11.72, and 10.12 mg/g, respectively. In the total pool of protein amino acids, the content of dietarily essential amino acids in the fruits of the analyzed cultivars ranged from 36% in ‘Glen Ample’ to 42% in ‘Radziejowa’. This group of amino acids was dominated by leucine (from 5.8% in ‘Glen Ample’ to 10.5% in ‘Radziejowa’), arginine (from 5.1% in ‘Glen Ample’ to 6.6% in ‘Laszka’), and phenylalanine (from 4.9% in ‘Radziejowa’ to 5.9% in ‘Glen Ample’). The level of non-essential amino acids was in the range from 58% in ‘Radziejowa’ to 64% in ‘Glen Ample’. The highest concentrations were determined in the case of aspartic acid (from 17.5% in ‘Radziejowa’ to 24.8% in ‘Glen Ample’), glutamic acid (from 15.5% in ‘Glen Ample’ to 17.2% in ‘Radziejowa’), and alanine (from 7.2% in ‘Radziejowa’ to 8.4% in ‘Laszka’) (Figure 5).

### 2.7. Content of Fatty Acids

**Figure 6:**
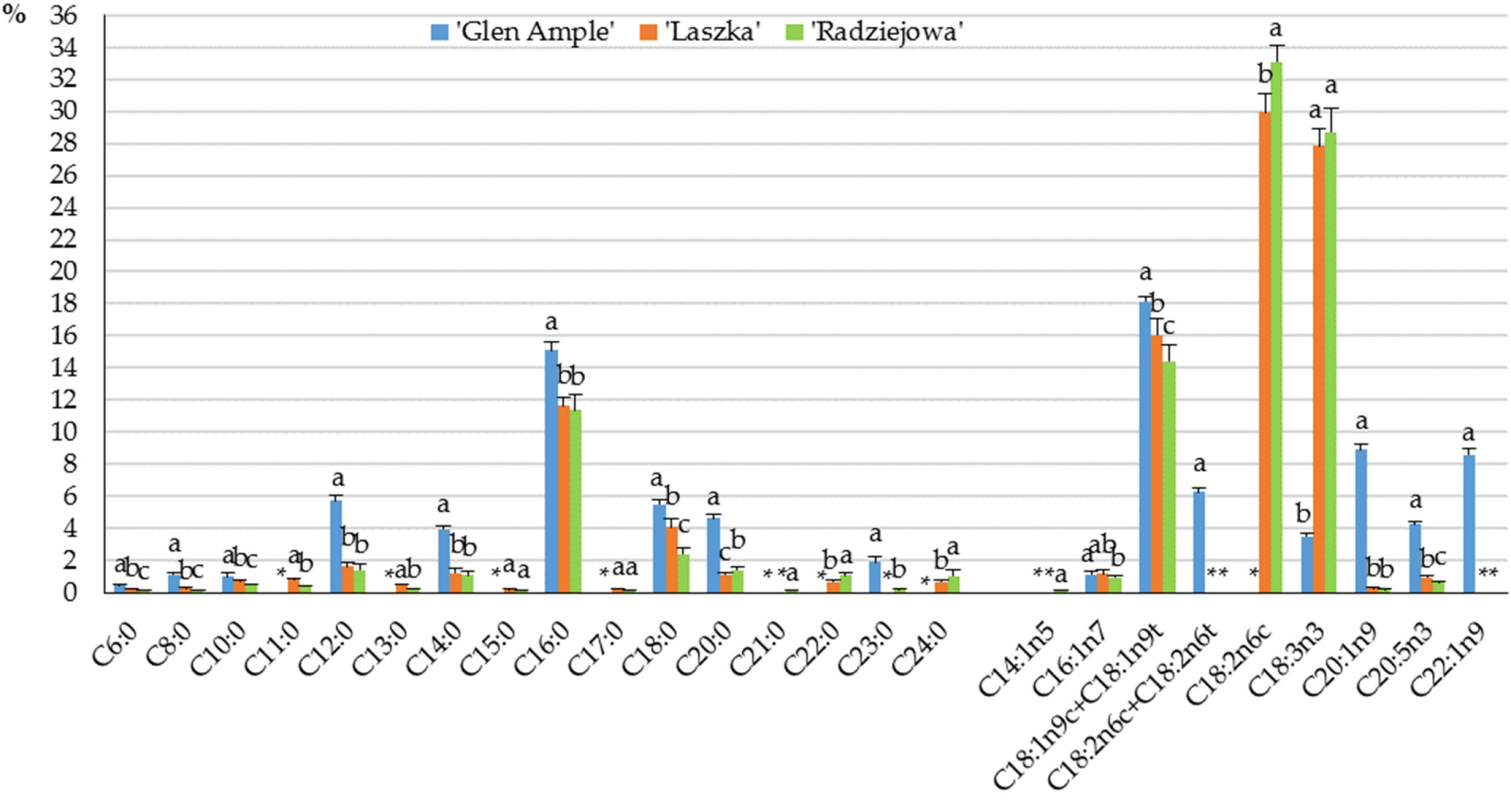
Contents of saturated fatty acids (SFAs) and unsaturated fatty acids (UFAs) in the fruits of biennial fruiting *R. idaeus* cultivars: Contents of saturated fatty acids (SFAs) and unsaturated fatty acids (UFAs) in the fruits of biennial fruiting *R. idaeus* cultivars: ‘Glen Ample’, ‘Laszka’, ‘Radziejowa’. Notes: C6:0 (caproic acid), C8:0 (caprylic acid), C10:0 (capric acid), C12:0 (lauric acid), C14:0 (myristic acid), C16:0 (palmitic acid), C18:0 (stearic acid), C20:0 (arachidic acid), C23:0 (tricosanoic acid), C16:1n-7 (palmitoleic acid), C18:1n9c (oleic acid), C18:1n9t (elaidic acid), (C18:2n6c gamma) gamma-linoleic acid, C18:2n6t (linoelaidic acid), C18:3n3 (alpha-linolenic acid), C20:1n9 (11-eicosenoic acid), C20:5n3 (eicosapentaenoic acid), C22:1n9 (erucic acid), not detected (*). Means of a single fatty acid followed by the same letter do not differ between the cultivars at a significance level of α = 0.05.

The fruits of the three *R. idaeus* cultivars contained sixteen saturated fatty acids (SFAs) and nine unsaturated fatty acids (UFAs). The total fatty acid sums in the *R. idaeus* ‘Glen Ample’, ‘Laszka’, and ‘Radziejowa’ fruits were 45, 68, and 104 mg/100 g f.w., respectively. In the group of long-chain SFAs, palmitic acid dominated with percentage contents of 18.4, 30.0, and 33.1% in the ‘Glen Ample’, ‘Laszka’, and ‘Radziejowa’ fruits, respectively. The other most abundant medium-chain fatty acid was lauric acid in ‘Glen Ample’ (5.7%), and the most abundant long-chain fatty acid was stearic acid in ‘Laszka’ and ‘Radziejowa’ (4.1 and 2.4%, respectively) (Figure 6).

In the group of UFAs, the highest concentrations were determined for γ-linoleic acid (C18:2n6c gamma)—29.95% in ‘Laszka’ and 33.1% in ‘Radziejowa’—and α-linolenic acid (C18:3n3 alpha) in the fruits of ‘Laszka’ (27.9%) and ‘Radziejowa’ (28.7%). In turn, the amounts of oleic acid (C18:1n9c) and elaidic acid (C18:1n9t) (18.4%) and 11-eicosenoic acid (C20:1n9) (8.9%) were dominant in the ‘Glen Ample’ fruits (Figure 6).

**Figure 7:**
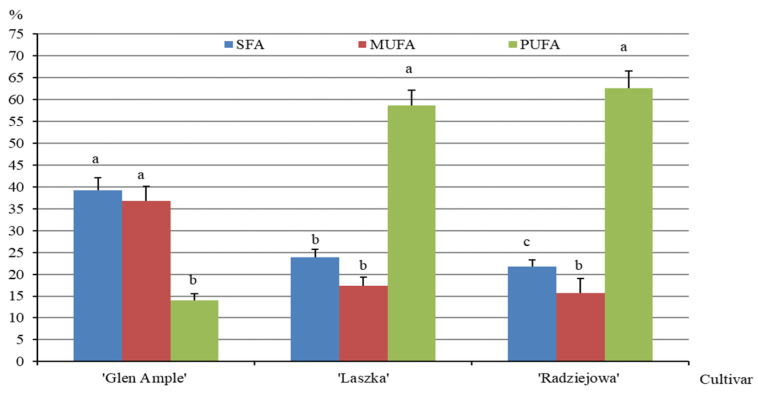
Percentages of saturated fatty acids (SFAs), monounsaturated acids (MUFAs), and polyunsaturated fatty acids (PUFAs) in the *R. idaeus* fruits. Notes: Means followed by the same letter do not differ between the cultivars at a significance level of α = 0.05. Vertical bars represent the standard deviations of the means.

With respect to the total pool of fatty acids, SFAs in the *R. idaeus* fruits constituted from 21.8% in ‘Radziejowa’ to 39.3% in ‘Glen Ample’. In turn, monounsaturated fatty acids (MUFAs) accounted for 15.6, 17.4, and 36.7% in the fruits of ‘Radziejowa’, ‘Laszka’, and ‘Glen Ample’, respectively. The content of polyunsaturated fatty acids (PUFAs) in the fruits of the analyzed cultivars ranged from 14% in ‘Glen Ample’ to 62.6% in ‘Radziejowa’. The SFA content in the fruits of ‘Laszka’ was significantly higher than in ‘Radziejowa’ and lower than in ‘Glen Ample’. The SFA contents in ‘Laszka’ and ‘Radziejowa’ did not differ significantly but were markedly below the value of this parameter recorded in ‘Glen Ample’. The level of PUFAs showed an opposite tendency to that of the MUFAs (Figure 7).

**Figure 8:**
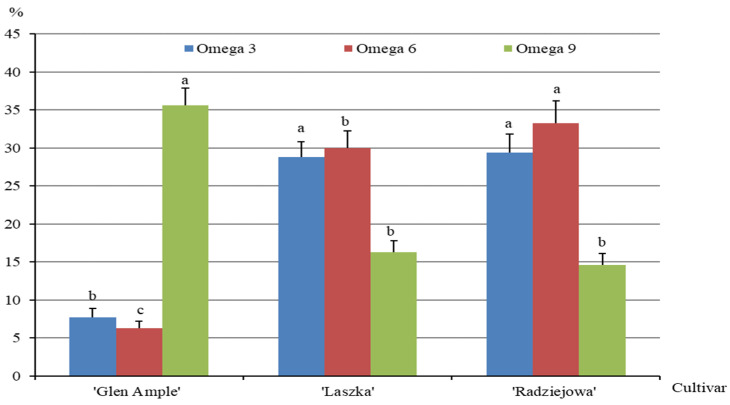
Percentages of omega 3, omega 6, and omega 9 fatty acids in the fruits of biennial fruiting *R. idaeus* cultivars. Notes: For each fatty acid group, means followed by the same letter do not differ between the cultivars at a significance level of α = 0.05. Vertical bars represent the standard deviations of the means.

In the group of PUFAs, acids from the omega 3, omega 6, and omega 9 families were detected in the fruits of the analyzed cultivars. The contents of omega 3 acids in ‘Glen Ample’, ‘Laszka’, and ‘Radziejowa’ were 7.7, 28.8, and 29.3%, respectively. This group was represented by alpha-linolenic acid (C18:3n3 alpha) and cis-5,8,11,14,17-eicozapentaenoic acid (C20:5n3). These acids were most abundant in the fruits of ‘Radziejowa’ (28.6%) and ‘Laszka’ (4.3%). Omega 6 fatty acids were represented by gamma-linoleic acid (C18:2n6c gamma) in the fruits of two cultivars: 6.3% in ‘Laszka’ and 30% in ‘Radziejowa’. In turn, the presence of the following omega 9 acids was detected: oleic acid (C18:1n9c), elaidic acid (C18:1n9t), and cis-11-eicosenoic acid (C20:1n9). The highest contents of the sum of acids (C18:1n9c + C18:1n9t) and C20:1n9 were found in the fruits of ‘Glen Ample’ (18.1%) and ‘Laszka’ (8.9%), respectively. The content of omega 3 acids in the fruits of ‘Glen Ample’ was significantly higher than in ‘Laszka’ and ‘Radziejowa’, and the opposite trend was recorded for the omega 9 content. The omega 6 level in ‘Laszka’ markedly exceeded that in ‘Glen Ample and was significantly lower than in ‘Radziejowa’ (Figure 8).

## 3. Discussion

### 3.1. Polyphenolic Compounds and Antioxidant Activity

The comparative methods used in the study, i.e., the FRAP, DPPH, Folin–Ciocalteau reagent, ABTS, and OH^•^ scavenging assays, confirmed the high free radical scavenging capacity of the fruits and fresh juice from *R. idaeus* ‘Glen Ample’, ‘Laszka’, and ‘Radziejowa’. The antioxidant activity of the analyzed cultivars was similar to the reducing power determined in *R. idaeus* ‘Heritage’, *R. innominatus*, and *R. niveus*, but lower than in *Rubus cyri*, *R. insularis*, and *R. caucasicus* × *Chester* [58,60]. Fruits of species of the genus *Rubus* are an excellent source of nutrients characterized by a high content of polyphenols. *Rubi idaei fructus* contains large amounts of these compounds, e.g., flavonoids and phenolic acids [61].

The average range of the total phenolic content in the *R. idaeus* fruits determined with the Folin–Ciocalteau method in the present study (1.0 mg/g in ‘Glen Ample’—1.3 mg/g in ‘Radziejowa’) was in agreement with the range reported for the raspberry cultivars ‘Aksu Kırmızısı’ (1.0–1.8 mg/g), ‘Rubin’ (1.1–1.9 mg/g), ‘Heritage’ (1.3–1.9 mg/g), and ‘Autumn Bliss’ (1.1–2.5 mg/g), but lower than the values reported for *R. idaeus* ‘Newburgh’ (1.4–1.8 mg/g), Heritage (1.5–1.9 mg/g), ‘Hollanda Boduru (1.8–2.1 mg/g) [62], ‘Fallgold’ (1.5–1.5 mg/g), ‘Meeker’ (2.1 mg/g) [63], and cloudberry *R. chamaemorus* L. (7.0 mg/g) [64]. These differences are mainly related to environmental factors. Since the total phenolic content in the studied raspberry cultivars is comparable to the antioxidant activity determined by the DPPH and ABTS assays, it may be supposed that the antioxidant activity of fruits is mainly related to the presence of polyphenols. Faleva et al. [64] have reported that the antioxidant activity of raspberry fruit extracts depends on the high content of ascorbic acid.

The average range of antioxidant activity measured with the different assays in the fresh fruits of the analyzed raspberry cultivars was from 8.3 in ‘Laszka’ to 9.7 umol/g in ‘Radziejowa’ (FRAP), 2.3 in ‘Glen Ample’ to 3.2 mg TE/g in ‘Radziejowa’ (ABTS), 1.1 in ‘Glen Ample’ to 1.3 mg TE/g in ‘Radziejowa’ (DPPH), and 17.9 in ‘Laszka’ to 19.5 mg TE/g in ‘Glen Ample’ (OH^•^ scavenging activity). The effect of antioxidants on DPPH scavenging is associated with their hydrogen-donating ability. DPPH is a stable free radical that accepts an electron or a hydrogen radical to become a stable diamagnetic molecule. The antioxidant activities (DPPH) of fresh raspberry fruits of different cultivars were in the following ranges: 76.6–122.4 μmol TE/g in ‘Aksu Kırmızısı’, 89.1–121.9 μmol TE/g in ‘Newburgh’, 64.1–96.7 μmol TE/g in ‘Rubin’, 66.0–81.2 μmol TE/g in ‘Heritage’, and 77.6–127.6 μmol TE/g in ‘Hollanda Boduru’. In turn, the results of the comparative analysis of the oxidative activity of fruits of these cultivars carried out with the ABTS method were as follows: 68.0–86.7 μmol TE/g, 83.7–98.6 μmol TE/g, 64.4–72.9 μmol TE/g, 65.4–74.3 μmol TE/g, and 69.7–117.1 μmol TE/g [62]. Mîrza [50] reported that the antioxidant activities of *Rubus* sp. fruit extracts determined with the ABTS assay were 13.9, 1.5, and 15.5 µM TE/g DW in *R. idaeus*, *R. fruticosus,* and *R. loganobaccus* ‘Tayberry Medana’, respectively. In turn, the oxidative activity in *R. idaeus* fruits of 14 genotypes and two cultivars, ‘Heritage’ and ‘Tulameen’, determined by Cekiç and Özgen [65] with the FRAP assay ranged from 9.8 to 11.2 μmol TE/g f.w.

The content of phenolic compounds in the juice from the fruit of the examined *R. idaeus* cultivars, ‘Glen Ample’, ‘Laszka’, and ‘Radziejowa’, was higher than their concentration in the fruit (21–49%). In turn, compared to the fruits, the antioxidant activity of the raspberry juice of the three cultivars determined with the ABTS, DPPH, FRAP, and OH^•^ scavenging assays was stronger by 1.5 (except for the ‘Radziejowa’ juice), 1.7–2.5, 2.5–3.5, and 3.7–4.2 times, respectively. Szymanowska et al. [52] reported the antioxidant activity of *R. idaeus* fruit juice at a level of 183.0 μM TE/100 g (f.w.), 166.3 μM TE/100 g (f.w.) in the phenolic fraction, and 107.8 μM TE/100 g (f.w.) (DPPH) in the anthocyanin fraction. The antioxidant activity in raspberry fruits and juice is mostly associated with their content of polyphenols (approximately 60%). The main mechanism of the antioxidant effect of juice consists in its chelating properties [66]. The dominant bioactive antioxidant compounds in *R. idaeus* juice are cyanidin derivatives, ellagic acid, and catechin, which are present in high concentrations [67]. Various fractions of *R. idaeus* fruit juice have health-promoting properties, e.g., they prevent imbalance between the production of free radicals influencing metabolic pathways and enzymes and cellular receptors [68].

### 3.2. Protein

The protein content in the fruits of the examined cultivars was higher in ‘Glen Ample’. Its amount in the studied cultivars, ‘Glen Ample’, ‘Laszka’, and ‘Radziejowa’, was higher than the range of 0.3–1.3% for this parameter in *R. idaeus* fruits described in the literature. Consumption of 250 g of *R. idaeus* fruit provides 3.1% of the daily protein requirement (75 g) [69]. Cross-reacting proteins with a complex IgE-reactivity pattern have been identified in *R. idaeus* fruits. At the DNA level, the PR-10 and PR-14 proteins (Rub i 1 and Rub i 3) exhibit the highest similarity to strawberry Fra a 1 and Fra a 3 sequences. Raspberries contain additional putative allergens, e.g., class III acidic chitinases and cyclophilins [70]. The Rub i 1 and Rub i 3 allergens in *R. idaeus* fruits exhibit similar sequences to the proteins of other taxa representing the family Rosaceae (Mal d 1 and Mal d 3 in apples, Pru av 1 and Pru av 3 in cherries, and Pru p 1 and Pru p 3 in peaches). Other proteins exhibit sequence homology with class III chitinases (raspberry chitinase). An IgE-reactive raspberry cyclophilin homologous to Bet v 7 has been identified, which suggests the presence of immunogenic cross-reacting proteins important in the diagnosis of cross-allergy. Diets containing raspberry fruit should take into account the possibility of development of an allergic reaction, and a higher level of anthocyanins may be an indicator of the concentration of allergic proteins [71]. Dietary supplementation with raspberry fruit increases the level of cytochrome C protein in the skeletal system, while the activation of protein kinase in skeletal muscles alleviates metabolic syndromes associated with obesity and insulin resistance [72,73,74].

### 3.3. Amino Acids

In the present study, the highest amino acid contents in the *R. idaeus* fruits were determined for leucine and arginine from the group of amino acids essential to humans, as well as aspartic and glutamic acids from the group of non-essential amino acids. Leucine represents the group of branched-chain amino acids (BCAAs); together with isoleucine and valine, they constitute approximately 35% of essential amino acids [75,76]. These amino acids stimulate glucose transport [77,78] and regulate gene expression and hepatocyte cell-cycle pathways [79,80]. The other dominant amino acid in the present study was arginine. L-arginine is a nitric oxide (NO) precursor [81,82]. In the present study, the group of endogenous acids was dominated by glutamic and aspartic acids. These amino acids are the main excitatory neurotransmitters in the central and autonomic nervous systems [83,84].

### 3.4. Vitamin C

The content of vitamin C in the fruits of the analyzed cultivars ranged from 28.2 in ‘Laszka’ to 33.6 mg/100 g f.w. in ‘Radziejowa’, and these values were within the concentration range of this compound in *R. idaeus* L. fruits (5–40 mg/100 g f.w.) described in the literature [33,69]. In turn, these values were higher than the content of ascorbic acid determined in the fruits of *R. idaeus* ‘Kweli’ (17 mg/100 g f.w.) and lower than the levels detected in ‘Fertodi’, ‘Meeker’, ‘Polka’, and ‘Willamette’ (35.8–54.9 mg/100 g f.w.) [85,86]. The ascorbic acid content in the fruits of different *Rubus* species reported by Schulz et al. [53] varied (mg/100 g f.w.) and was 92.2 in *R. idaeus*, 29.8 in *R. ellipticus*, and in the range of 7.1–10.6 in five other species: *R. niveus*, *R. ulmifolius*, *R. fruticosus*, *R. adenotrichus*, and *R. glaucus.*

### 3.5. Flavonoids

In the present study, the total content of flavonoids in the fruits of the analyzed cultivars was 2.2–3.6 mg/100 g (calculated as rutin). The content of flavonoids in the fruits of 18 *Rubus* cultivars varied and ranged from 6 mg/100 g to 46.9 mg/100 g (calculated as rutin), and no flavonoids were detected in two cultivars [69]. In turn, this parameter in the fruits of *R. ellipticus*, *R. fairholmianus*, and *R. niveus* was in the range of 185–215 mg RE/g (calculated as rutin) [87]. The difficulty in the comparison of the results of the total flavonoid content in raspberry fruits described in the literature is associated with the different methodological techniques used in various studies [52,62,88]. Sariburun et al. [62] showed a strong correlation of the total flavonoid content with the ABTS value and a low correlation of the flavonoid content with DPPH. Flavonoids have the ability to transfer electrons to free radicals, chelate metal catalysts, activate antioxidant enzymes, and mitigate nitric oxide-induced oxidative stress [50]. The concentrations of flavonoids in fruits are determined by genetic and many environmental factors (soil, cultivation, irrigation, and climatic conditions) that may result in the variability in the composition of secondary metabolites in the total yield. The biosynthesis of flavonoids in plants is positively influenced by long and cool days and increased UV irradiation, with differences between species and groups of these compounds. The regulation of the flavonoid biosynthesis process depends on signals transmitted in response to environmental factors to various tissues during development processes [89]. Phenolic acids protect against insects and microorganisms as well as lipid oxidation in fruits; hence, the content of these compounds depends on the development stage. A higher content of phenolic compounds was determined in unripe versus ripe fruits and in fruits from organic (flavonoids and phenolic acids) versus conventional cultivation. The phenolic content may be modified by oxidation taking place during processing and storage [90].

### 3.6. Anthocyanins

The content of anthocyanins in the analyzed *R. idaeus* fruits ranged between 22 mg/100 g f.w. in ‘Glen Ample’ and 34 mg/100 g f.w. in ‘Laszka’. These values either coincided with the range of this parameter described in the literature for raspberry fruits, i.e., 0.2–100.0 mg/100 g [33,69], or were lower than the concentrations in the fruits of *R. idaeus* ‘Fertodi’, ‘Meeker’, ‘Polka’, and ‘Willamette’ (56.8–58.8 mg/100 g) and in 12 other cultivars, ‘Anitra’, ‘Fertodi’, ‘Glen Ample’, ‘Glen Carron’, ‘Glen Fyne’. ‘Meeker’, ‘Ninni’, ‘Polka’, ‘Tulameen’, ‘Varnes’, ‘Veten’, and ‘Willamette’ (34.5–70.8 mg/100 g) [85,91]. The value of this parameter in fruits of several other species of the genus *Rubus*, i.e., *R. idaeus*, *R. laciniatus*, *R. occidentalis*, *R. ursinus*, and *R. ursinus × idaeus*, ranged from 0.7 to 5.9 mg/g f.w. [92]. The content of anthocyanins in different-colored *Rubus* species was reported to range from 2.1 (yellow fruits) to 325.5 mg/100 g (black fruits) [93].

The daily intake of anthocyanins has been estimated at 12.5 mg/day/person, with fruits and berries recommended to account for 70% of the total intake [94,95]. The daily recommended intake of anthocyanins by an adult is 50 mg [96]. The daily consumption of anthocyanins should range from a few to hundreds of milligrams, depending on requirements, eating habits, and individual preferences. To maintain a healthy lifestyle, 3.0–215.0 mg of anthocyanin-containing foods should be consumed daily [97]. Anthocyanins are essential dietary nutrients, as they reduce levels of oxidative stress and, consequently, the risk of cancer, metabolic syndrome, diabetes, and degenerative diseases [96]. It has been estimated that daily consumption of 100 g of raspberry fruit with the average anthocyanin content of 37.5 mg/100 g covers 75% of the daily requirement for these compounds [70]. The anthocyanin contents calculated for 100 g of fruits of the examined *R. idaeus* cultivars, ‘Glen Ample’, ‘Radziejowa’, and ‘Laszka’, were 39.4, 48.4, and 67.8, respectively.

### 3.7. Fatty Acids

The group of UFAs contained in the analyzed *R. idaeus* fruits was dominated by gamma-linoleic acid (C18:2n6c gamma) and alpha-linolenic acid (C18:3n3). They were the most abundant in the ‘Radziejowa’ fruits, i.e., 31% and 28.7%, respectively. In the group of SFAs, the highest concentrations were determined for palmitic (C16:0) (18–33%), lauric (C12:0) (6%), and stearic (C18:0) (2–4%) acids. The SFAs, MUFAs, and PUFAs in the fruits of the analyzed *R. idaeus* cultivars accounted for 22–39, 16–37, and 14–63%, respectively. These values were similar to or higher than those described in another experiment on raspberry fruits (46% (SFAs), 22.7% (MUFAs), and 31.3% (PUFAs)) [98]. In a study conducted by Celik and Ercisli [99], the contents of these groups of fatty acids in the raw material were in the ranges of 6–9.7, 12.1–17.1, and 67–75.9%, respectively. In the present study, the lipid fraction in the *R. idaeus* fruits was rich in UFAs, especially PUFAs, which are indispensable for the structure of cell membranes, nerve protection, proper blood coagulation, muscle movement, protection against inflammation, and proper functioning of the organism [22]. Dietary intake of PUFAs, e.g., omega 3, and MUFAs, given their cardioprotective role, is recommended during a pandemic or in circulatory system diseases. A diet rich in essential unsaturated fatty acids supports vasodilation and has anti-inflammatory, antiarrhythmic, antithrombotic, antioxidant, and antiatherosclerotic effects [100]. Similarly, raspberry seed oil is used as a pro-health dietary supplement, as it contains a favorable ratio of omega-6 to omega-3 fatty acids (1.4:1) [101].

In the present study, the contents of omega 3, omega 6, and omega 9 acids in the fruits of the *R. idaeus* cultivars were in the ranges of 7–29.3, 6.3–33.2, and 14.6–35.6%, respectively. As recommended by the European Scientific Committee on Food (ESCF), 2% and 0.5% of the total daily energy intake should be provided by omega-6 and omega-3 fatty acids, respectively [101]. In turn, the World Health Organization (WHO) recommends the intake of 2.5–9% of omega-6 fatty acids and 0.5–2% of omega-3 fatty acids in the daily energy pool. According to the Food and Agriculture Organization and the WHO, from 2 to 4% of daily energy should be provided by essential fatty acids, with an additional 3% recommended for pregnant or breastfeeding females [22]. The omega 3 group detected in the present study was dominated by alpha-linolenic acid (C18:3n3) and cis-5,8,11,14,17-eicosapentaenoic acid (C20:5n3). In turn, gamma-linoleic acid (C18:2n6c gamma) was the most abundant omega 6 acid, and oleic C18:1n9c), elaidic (C18:1n9t), and cis -11-eicosene (C20:1n9) acids dominated in the omega 9 group. Reports in the literature confirm the dominant content of these fatty acids in *R. idaeus* fruits. All these acids have a pro-health effect, and omega-3 and omega-6 are crucial in the phytotherapy of the cardiovascular system and diabetes [22]. Furthermore, they reduce UV-induced inflammation [21,102]. They are also involved in the structure of cell membranes and the synthesis of intracellular lipids in the stratum corneum, regulating their elasticity [103]. A diet rich in fatty acids and low in dietary fiber increases the risk of obesity, type 2 diabetes, and cardiovascular diseases [101].

### 3.8. Fiber

The content of fiber in the fruits of the *R. idaeus* cultivars ranged from 4.7 to 6.1%, and these values were within the range (2–6.5%) for raspberry fruits described in the literature [69,104,105]. Fiber in raspberry fruits consists mainly of insoluble compounds, primarily hydrolyzing polyphenols. The insoluble dietary fiber fraction is rich in phenolic compounds and has antioxidant and fat-retention properties. In turn, soluble fiber has the ability to swell, retain fat and water, and delay glucose diffusion. Raspberry fiber fractions can be used as functional and prebiotic ingredients in food products with enhanced physical and nutritional properties [106]. Consumption of raspberries can enhance the detoxification defenses of cells due to their content of fiber. Raspberry fiber protects against oxidative stress associated with obesity and hyperglycemia. It also improves antioxidant activity and reduces interleukin (IL)-6 levels, most probably through enhancement of glutathione peroxidase activity in the liver and blood [105]. 

### 3.9. Application

Given the growing consumer awareness of natural pro-health bioactive chemical compounds, the fruits and juice of *R. idaeus* ‘Glen Ample’, ‘Laszka’, and ‘Radziejowa’ can be rich sources of antioxidants and antioxidant activity, as confirmed by the FC, DPPH^•^, ABTS^•+^, TPH, FRAP, and OH^•^ scavenging assays. Regular consumption of raspberry fruit helps to prevent many diseases. With the nutritional properties and antioxidant activity determined in the present study, the analyzed raw material can be used in the food, pharmaceutical, and cosmetic industries. Currently, many studies are focused on the search for natural active phytochemicals that can be used in the development of new products supporting the therapy of many diseases. The improvement of methods for the manufacture of juices and drinks characterized by high antioxidant activity is gaining increasing interest. Therefore, the present study may help producers and consumers to understand the importance of traditional nutraceutical products based on fresh or freeze-dried *R. idaeus* fruits characterized by high nutritional and antioxidant values. The fruits of the analyzed *R. idaeus* cultivars can be a source of active ingredients for biopharmaceutical and biocosmetic engineering and for the manufacture of food and nutraceutical products. They can also be recommended as an inexpensive, high-quality, and health-enhancing ingredient in the daily diet.

### 3.10. Future Research

In further research, *R. idaeus* fruits may be investigated as a commercially valuable raw material to be used in innovative programs and projects focused on progress in molecular genetics, genomics, and metabolomics as well as supramolecular chemistry combined with medical biophysics. To meet consumers’ expectations, it is important to understand and use biochemical pathways with appropriate co-linearity in genetic engineering to design new raspberry cultivars with high fruit quality, a rich composition of active phytochemicals, and nutritional and phytotherapeutic properties ensuring better dietary and pro-health effects, e.g., immunological and metabolic outcomes achieved through appropriate nutritional intervention.

## 4. Materials and Methods

### 4.1. Study Material

*Rubus idaeus* L. fruits were collected for the analyses from a plantation located in Blinów II, Lublin Province, south-eastern Poland (50°52′57.03″ N; 22°23′2.663″ E). Samples of ripe and well-formed ‘Glen Ample’, ‘Laszka’, and ‘Radziejowa’ fruits (biennial fruiting cultivars) were collected to determine the contents of selected active chemical compounds. The fruits of the three cultivars were collected at harvest ripeness at the turn of the third ten days of June and at full harvest ripeness on the first ten days of July. In order to obtain natural fruit juice, fruits samples (500 g) were processed in a juice extractor (Juicer, Philips HR 1853). Juice replicates were centrifuged at 4000 rpm for 1 h and subjected to analysis at the day of preparation.

#### Origin of Cultivars

The ‘Glen Ample’, ‘Laszka’, and ‘Radziejowa’ biennial fruiting cultivars of *R. idaeus* are commonly cultivated in commercial plantations. ‘Radziejowa’ and ‘Laszka’ are Polish cultivars. They were developed in the Experimental Station of the Research Institute of Horticulture in Brzezna by Agnieszka Orzeł, PhD, and Jan Danek, PhD [107]. *R. idaeus* ‘Radziejowa’ was created by crossing two clones, nos. 92271 and 96221. The lineage of the ‘Radziejowa’ cultivar includes the cross of the Polish cultivars ‘Laszka’ and ‘Polana’ with the English ‘Malling Promise’ and American ‘Canby’ cultivars. On 25 January 2010, the ‘Radziejowa’ cultivar was registered in the National Register of Horticultural Plant Varieties kept by the Central Research Center for Crop Plant Varieties. The cultivar ‘Laszka’ was developed by crossing clones 80408 × 80192 in the Experimental Station of the Research Institute of Horticulture in Brzezna. On 8 February 2006, it was listed in the National Register of Horticultural Plant Varieties [107,108]. The cultivar ‘Glen Ample’ is a licensed Scottish variety developed in 1978 at the Scottish Institute of Plant Production in Dundee, UK. It was created by crossing clones SCRI7336E1 X SCRI7412H16. Its lineage also includes the cross of the English ‘Glen Prosen’ × American ‘Meeker’ [107,109,110].

### 4.2. Determination of Antioxidant Activity

Antioxidant activity and total polyphenol content were determined in fresh mature healthy raspberry fruits with an appropriate shape and color and in the fruit juice. Samples of blended fruits and juice were extracted in a MARS 5 microwave extractor (Varian) with the use of 100 mL of water at 100 °C for 10 min with the device power set at 800 W. The fruit extract was filtered, and an appropriate amount was used in the subsequent analyses. The following methods were employed to determine the antioxidant potential: Folin–Ciocalteau (FC), ferric reducing antioxidant power (FRAP), 2,2-diphenyl-1-picrylhydrazyl (DPPH), 2,2′-azinobis-(3-ethyl-benzothiazoline-6-sulfonic acid) (ABTS), and OH^•^ radical scavenging assays. Total polyphenols were determined with the spectrophotometric method using Folin–Ciocalteau (FC) reagent.

#### 4.2.1. FRAP Method (Ferric Reducing Antioxidant Power Assay)

The antioxidant activity of the *Rubi fructus* raw material was assessed using the ferric reducing antioxidant power (FRAP) assay [111]. A quantity of 3.6 mL of the FRAP reagent was added to 0.2 mL samples of the water-filtered fruits and fruit juice extracts and made up to a volume of 25 mL with distilled water. The absorbance of the solutions was measured at a wavelength of λ = 593 nm using a Hitachi U-2900 spectrophotometer (Hitachi, Tokyo, Japan). A standard curve was plotted for iron (II) sulfate (VI) concentrations: 10, 15, 20, 40, and 50 µM (y = 0.0205x, r = 0.998), and the absorbance values were converted into FRAP units, which expressed the amount of antioxidant compounds per 1 g of raw material (µmol/g) or 1 mL juice (µmol/mL) capable of reducing 1 mole of iron(III) to iron(II). The FRAP reagent was prepared by mixing together 10 mM of 2,4,6-Tripyridyl-S-triazine (TPTZ), 20 mM of FeCl_3_, and 300 mM of acetate buffer, pH 3.6, at a ratio of 10:1:1.

#### 4.2.2. DPPH Method

The antiradical activity of the *Rubi fructus* raw material was determined as in Brand-Williams et al. [112] and Bondet et al. [113] with the use of the 2,2-diphenyl-1-picrylhydrazyl (DPPH) radicals. A quantity of 3.9 mL of a methanol solution of the DPPH radicals (3.9 mL) at a concentration of 6 × 10^−5^ M was added to 0.1 mL of the samples of the water-filtered fruits and fruit juice extracts. After 30 min, the decrease in the absorbance of the solution was measured for 12 min with a time interval of 60 s at a wavelength of λ = 515 nm (maximum absorbance of DPPH^•^ radicals) until a state of equilibrium was reached. The absorbance result was converted into a Trolox unit based on the standard curve. The results were expressed as the content of antioxidant compounds capable of reducing DPPH^•^ radicals in 1 g of raw material or 1 mL of juice equal to 1 mg of Trolox (6-Hydroxy-2,5,7,8-tetramethylchroman-2-carboxylic acid).

#### 4.2.3. ABTS Method

The antioxidant activity was determined using 2,2′-azinobis-(3-ethyl-benzothiazoline-6-sulfonic acid) (ABTS) radicals as in Re et al. [114]. A 30 µL sample of the extract was supplemented with 3 mL of an aqueous solution of the ABTS radicals with an approx. 0.7 absorbance value. After one hour, the decrease in absorbance was measured at 734 nm using a Hitachi U-2900 spectrophotometer (Hitachi, Tokyo, Japan). The results were expressed as the amounts of compounds contained in 1 g of the raw material equivalent to Trolox units (vitamin A derivative, 6-hydroxy-2,5,7,8-tetramethylchroman-2-carboxylic acid).

#### 4.2.4. OH^•^ Radical Scavenging Method

The OH^•^ radical scavenging method was used as in Gawlik-Dziki et al. [59]. The technique consists of the generation of OH^•^ radicals via the Fenton reaction. The radicals were generated by mixing appropriate amounts of iron sulfate and hydrogen peroxide. Appropriate amounts of the water-filtered fruits and fruit juice extracts and sodium salicylate were added to the reaction mixture. The antioxidant compounds contained in the raw material reduced the generated radicals, and the excess reacted with sodium salicylate. The degree of the reaction of the raw material with the radicals was inversely proportional to the pink color of the reaction mixture, which showed absorbance at the 562 nm wavelength. The measurement results were converted into Trolox equivalents, and the antioxidant activity of the samples was expressed as the amount of antioxidant compounds per mg of TE contained in 1 g of the raw material.

#### 4.2.5. Folin–Ciocalteau Method

The total content of phenolic compounds was determined with the Folin–Ciocalteau reagent method as in Singleton and Rossi [115] and Prior [76]. After filtering the fruit and fruit juice extracts with water, 2 mL of Folin–Ciocalteau reagent was added to 1 mL of these extracts. After 3 min, the reaction environment was alkalized with 10 mL of 10% Na_2_CO_3_, and then after 30 min., the solutions were made up to a volume of 25 mL and their absorbance was measured at λ = 765 nm with a Hitachi U-2900 spectrophotometer (Hitachi, Tokyo, Japan). The results were expressed in mg of phenolic compounds per 1 g of raw material or in mg of phenolic compounds per 1 mL of juice as caffeic acid equivalents.

### 4.3. Chemical Analyses

The chemical composition of the fruits was analyzed to determine the content of available carbohydrates, total sugars, fiber, protein, fat, water, and ash. The water content was determined with the drying method using a Memmert oven. The ash content was determined in triplicate [116]. The samples were placed in a Czylok muffle furnace at 550 °C. They were incinerated until light-gray ash or a constant weight was obtained (7 h). After cooling, the samples were weighed, and the ash contents were calculated.

#### 4.3.1. Total Sugars

The total sugar content in the fruits of the analyzed cultivars was determined with the Luff–Schoorl method according to the Polish Standard PN-90/A-75101/07:1990 [117]. The method involves the reduction of Cu^2+^ ions contained in Luff’s solution by sugars present in a sample at pH = 9.5 at the boiling point. Unreduced copper ions are titrated with sodium tiosulfate (VI). The difference between the sodium thiosulfate (VI) utilized in the blank sample and in the reaction with saccharide-reduced copper(II) in the sample corresponds to the amount of copper reduced directly by the reducing sugars. Ten-gram samples were placed in a beaker and topped up with distilled water to a volume of 50 cm^3^. Next, Carrez solutions I and II were added, mixed, and topped up to a volume of 200 mL. Afterwards, 10 mL of the solution was taken, 25 mL of Luff’s solution was added, and the mixture was brought to boiling for 10 min. After cooling, 3 g of potassium iodide and 25 mL of sulfuric acid were added and titrated with a sodium thiosulfate solution until a light-yellow color was achieved. At the end, 1 mL of a starch solution was added. A similar procedure was employed for the blank sample, but 25 mL of water was added instead of the sample. The difference between the amount of thiosulfate utilized in the blank sample and the tested sample corresponded to the amount of copper reduced directly by reducing sugars. For sucrose inversion, 5 cm^3^ of HCl was added to 25 cm^3^ of the filtrate, heated in a water bath to 70 °C for 5 min, cooled rapidly, neutralized with a 30% NaOH solution, and topped up with distilled water to a volume of 100 cm^3^. Next, 25 mL of Luff’s solution was poured into a 250 mL conical Erlenmeyer flask, 10 mL of the analyzed solution was added, and distilled water was added to a volume of 50 mL. The mixture was brought to the boil for 2 min, and a gentle boiling process was maintained for 10 min. The solutions were cooled to 20 °C, 3 g of potassium iodide and 25 mL of a 25% sulfuric acid solution were added, and then the mixture was titrated with 0.1 M sodium thiosulfate. Finally, 2 mL of a 2% starch solution was added until a creamy color was visible. Simultaneously, a blank test with distilled water instead of the solution was performed.

#### 4.3.2. Total Fiber

The amount of fiber in the fruits was determined using the enzymatic method described by Asp et al. [118] in accordance with the PN-A-79011-15:1998 standard [119]. The sample was treated with α-amylase, pepsin, and pancreatin. The amount of undigested insoluble and soluble fiber precipitated from the filtrate was determined using the gravimetric method. Fruit samples were homogenized in 20 mL of sodium phosphate buffer (pH 6.0), followed by the addition of α -amylase [termamyl; 50 l] and incubation (30 min, 50 °C) with occasional stirring. After cooling the contents to room temperature, 10 mL of water was added and the pH was adjusted to 1.5 with 4 M HCl. Next, 50 mg of pepsin was added, and the mixture was incubated at 37 °C for 1 h in a shaking water bath. After recooling the contents to room temperature, 10 mL of water was added and the pH was adjusted to 6.8 with a 4 M NaOH solution. This mixture was supplemented with 50 mg of pancreatin and incubated for 1 h at 37 °C. The contents were cooled; the pH was adjusted to 4.5 with 4 M HCl and filtered through a dried and weighed crucible containing 0.5 g of celite. The drying and weighing were performed by adding 0.5 g of celite to the crucible and washing it with 20 mL of 95% ethanol and 20 mL of acetone. Then, it was dried in an oven at 105 °C for 30 min and weighed.

#### 4.3.3. Total Protein

The total nitrogen content in the fruit samples (n = 3) was determined with the micro-Kjeldahl method [120] using a FOSS Kjeltec 2300 nitrogen Auto analyzer (Foss Tecator, Höganäs, Sweden). The samples were completely digested in H_2_SO_4_-H_2_O_2_. The distillation and titration were automatically conducted by the Kjeldahl system. The total protein content was estimated following the Polish standard PN-75 A-04018:2002) [121] by multiplying the nitrogen content by a protein factor of 6.25.

#### 4.3.4. Amino Acids

The qualitative and quantitative composition of amino acids in the fruits was determined based on the acid hydrolysis of proteins as described by Davies and Thomas [122]. An aliquot of the ground sample (5 g) was placed in an INGOS hydrolyzer thimble (INGOS HBO 16, Prague, Czech Republic) and flooded with 6M HCl. The solution was saturated with nitrogen, hydrolyzed at 110 °C for 20 h, cooled, and filtered with a G5 filter funnel. The hydrolysate (4 mL) was evaporated in an RVO 400 SD vacuum evaporator (Hamburg, Boeco, Germany) at 50 °C, washed with 1 mL of distilled water, and evaporated again. The dry residue remaining in the vacuum flask was dissolved in 5 mL of citrate buffer, pH 2.2. The sample was added to a 35 cm long column with a 5 mm diameter filled with ion-exchange resin. After filtration through a syringe filter, the prepared sample was dispensed into the column of an AAA amino acid analyzer (Ingos, Praque, Czech Republic).

The hydrolysis of proteins for the separation of sulfuric amino acids was performed as in Schramm and Moor [123]. Cysteine was oxidized to cysteic acid, while methionine was oxidized to methionine sulfone by flooding the sample with performic acid and leaving it overnight in a freezer. The next day, the mixture was flooded with 40% HBr, stirred, and allowed to stand for several minutes. Next it was concentrated in an RVO 400 vacuum evaporator, flooded with 6N HCl, and transferred to an HBO 16 Ingos hydrolyzer thimble. The hydrolysis was carried out as described above.

In order to determine the tryptophan content, the ground sample was subjected to alkaline hydrolysis with 4.2M Ba(OH)_2_ at 110 °C for 20 h using an HBO 16 INGOS hydrolyzer. Subsequently, the sample was acidified with 6 N HCl, and a Na_2_SO_4_ solution was added in order to precipitate barium ions. The precipitated BaSO_4_ was centrifuged for 15 min at 3000 rpm using a Hettich ROTOFIX 32A benchtop centrifuge. After filtering through a syringe filter, the supernatant was loaded into an amino acid analyzer column.

Amino acids were separated using an AAA 400 automatic amino acid dose analyzer (Ingos, Prague, Czech Republic) at temperatures T1 = 60 °C and T2 = 63 °C. Amino acid identification using ion-exchange chromatography was carried out via a photometric detector (ninhydrin) at a wavelength of 570 nm for all amino acids and at 440 nm for proline. The separation was carried out in a 3.7 × 450 mm column packed with a strong cation exchanger Ostion LG ANG (INGOS company), with an average grain size of approx. 12 µm in the form of Na. The column temperature was 60–74 °C. In addition to the ninhydrin detector, the mobile phase consisted of the following set of four buffers with a pH gradient applied for separation: 1, pH 2.6; 2, pH 3.0; 3, pH 4.25; and 4, pH 7.9. After the amino acid separation, the column was regenerated with 0.2N NaOH. The chromatogram was read using the Chromulan computer program (version 0.9).

#### 4.3.5. Total Fat Content

The total fat content was estimated in accordance with the Polish Standard PN-EN ISO 5508:1996 [124]. The Soxhlet method based on acid hydrolysis of 4 M HCl was used in this analysis. The method involves liquid–solid solvent extraction. The samples were weighed into thimbles and placed in the extraction unit—a Soxtec Avanti^®^ (Tecator, Buchi, Switzerland). The extraction vessels were filled with the solvent, and the soluble material was extracted in a two-step process, followed by a solvent recovery phase. In the final step, the extraction vessels were dried (MEMMERT laboratory dryer, Schwabach, Germany) and weighed (PS 750/X Analytical Balance, Radwag, Radom, Poland). The fat content was expressed as a % based on the weight of the fat and the weight of the sample.

#### 4.3.6. Total Carbohydrate Content

A computational method was used to calculate available carbohydrates. The principle of this method is to calculate the total carbohydrate content after determination of the content of basic food ingredients, i.e., moisture, protein, fat, and total ash. The sum of carbohydrates complements the sum of the other chemical components to 100%.

#### 4.3.7. Qualitative and Quantitative Composition of Fatty Acids

The quantitative and qualitative analysis of fatty acids was performed in accordance with the Polish Standard PN-EN ISO 12966-1:2014 [125]. The saponification process was carried out using a methanolic solution of potassium hydroxide. Next, esterification was performed by the addition of a methanolic boron trifluoride solution, and separation was carried out using hexane and a saturated sodium chloride solution. The hexane layer was collected into a glass vial and dried over anhydrous sodium sulfate. The chromatographic analysis was performed using a Varian 450-GC gas chromatograph (Temecula, CA, USA) equipped with a 1177 Split/Splitless injector at a temperature of 250 °C with a Select™ Biodiesel for FAME capillary column (30 m; 0.32 mm; 0.25 μm). The stationary phase included Select Biodiesel for FAME Fused Silica, a column furnace with a starting temperature of 100 °C and a final temperature of 240 °C, and an FID detector (temperature of 270 °C). The carrier gas (helium) flow rate was 1.5 mL/min. Galaxie™ Chromatography Data System Autosampler CP-8400 software was used for the collection, integration, and calculation of results.

#### 4.3.8. Vitamin C

The content of vitamin C was determined by high-performance liquid chromatography (HPLC) according to the external standard PN-EN 14130:2004 [126]. The extraction of the ground fruit samples was carried out with the use of a metaphosphoric acid solution. In this process, dehydro-L(+) ascorbic acid is transformed into L(+) ascorbic acid. A 3 g fruit sample was treated with 80 mL of 3% metaphosphoric acid and shaken; after filtration, a 100 mL flask was filled with this acid. To reduce the resulting solution (20 mL), 10 mL of an L-cysteine solution was added, trisodium phosphate was used to achieve a pH = 7.0–7.2, and metaphosphoric acid was added. The total content of L-(+)- ascorbic acid was determined in 3 replications by reverse-phase HPLC (RP-HPLC) in a C18 column with UV detection at 265 nm. The limit of detection was 1.5 µg/cm^3^, and the limit of quantification was 5 µg/cm^3^. Column temperature: 22 °C; composition of the eluent: 95/5 CH_3_COOH: CH_3_OH; flow rate: 0.7 cm^3^/min^1^.

#### 4.3.9. Flavonoids

The total flavonoid content was determined with the method proposed by Lamaison and Carnet [127]. A 1 g fruit sample was transferred to a flask and 20 mL of acetone, 2 mL of hydrochloric acid, and 1 mL of methenamine solution were added. The mixture was kept under reflux in a boiling water bath for 30 min. The hydrolysate was filtered into a volumetric flask (100 mL) and supplemented with acetone. After transferring 20 mL of the solution into a separatory funnel, 20 mL of water was added, and the mixture was extracted with ethyl acetate in 15 mL portions and 3 times in 10 mL portions. The combined organic layers were washed with water (40 mL each) twice, filtered into a volumetric flask (50 mL), and supplemented with ethyl acetate. The solution intended for the analyses was prepared by the addition of 2 mL of an aluminum chloride solution (20 g/l) to 10 mL of the stock solution followed by the addition of an acetic acid:methanol mixture (1:19) to a volume of 25 mL. Next, the reference solution was prepared by supplementation of 10 mL of the stock solution with an acetic acid:methanol mixture (1:19) to 25 mL. After 45 min, the absorption of the solutions at 425 nm was measured and compared with the reference solution. The percentage of flavonoids was expressed as rutin equivalents.

#### 4.3.10. Anthocyanins

The accumulation of anthocyanins in the fruits was determined using the modified method proposed by Martinez [128]. Anthocyanins were extracted by maceration of fresh samples in a methanol:HCl solution (99:1, *v*/*v*) at 4 °C for 24 h. The extracts were centrifuged at 10,000 rpm for 10 min. at 4 °C, and their absorbances were read at 527 nm and 652 nm (Cecil CE 9500, Cecil Instruments, Cambridge, UK). The concentration of anthocyanins was calculated using the molar extinction coefficient (ε = 29,600 M^−1^ cm^−1^) for cyanidin 3-glycoside (C3G).

### 4.4. Energy Value

The energy value of the fresh fruits of the analyzed raspberry cultivars was determined according to the EU Commission Delegated Regulation No. 78/2014 [129].

### 4.5. Statistical Analysis of the Results

The means of the measurements and readings as well as the standard deviations were calculated. The significance of differences was analyzed statistically using Statistica 6.0 software. Differences between selected traits were assessed with the use of one-way ANOVA. Statistical inference was performed at a significance level of *p* < 0.05.

## 5. Conclusions

The antioxidant activity of raspberry fruit juice was 2.5 to 3.5 times higher in the FRAP method and 3.7 to 4.2 times higher in the OH hydroxyl radical assay compared to this parameter in the fruits. The other methods (DPPH and ABTS) used to determine antioxidant properties confirmed that the *Rubi idaei fructus* and juice from *R. idaeus* ‘Glen Ample’, ‘Laszka’, and ‘Radziejowa’ strongly reduced free radicals. The ‘Radziejowa’ fruits and the ‘Laszka’ juice were characterized by the highest antioxidant activity. Taking into account the content of polyphenolic compounds, the cultivars ranked as follows: ‘Glen Ample’ < ‘Laszka’ < ‘Radziejowa’ (fruits) and ‘Glen Ample’ < ‘Radziejowa’ < ‘Laszka’ (juice). The low energy value (39–47 kcal/100 g) of the fruits and their high content of dietary fiber indicate that they can be consumed by dieting subjects. Moreover, the high content of vitamin C (28.2–33.6 mg/100 g) and anthocyanins (19.7–33.9 mg/100 g) proves their high biological activity and health-promoting value. Palmitic and stearic acid predominated in the group of SFAs, while gamma linoleic acid (C18:2n6c gamma) (30–31%) and alpha-linolenic acid (C18:3n3 alpha) (27–27.9%), which neutralize excess free radicals, were the most abundant unsaturated fatty acids. The group of PUFAs was dominated by omega 6 acids, followed by omega 3 and omega 9. In the fruits of the analyzed raspberry cultivars, essential amino acids accounted for 36–42% of the total pool of amino acids, with the dominance of leucine (5.8–10.5%), arginine (5.1–6.6%), and phenylalanine (4.9–5.9%). To sum up, raspberry fruits and juice are a good source of antioxidants, which are important elements in the treatment of oxidative stress-related diseases. To the best of our knowledge, this is the first report comparing the antioxidant activity of fruits and juice and the content of bioactive compounds in the fruits of the *R. idaeus* ‘Glen Ample’, ‘Laszka’, and ‘Radziejowa’ cultivars, which are well-known and highly commercialized crops in Europe.

## Figures and Tables

**Table 1:** Total content of phenolic compounds (FC) and antioxidant activity (FRAP, DPPH, ABTS, and OH^•^ scavenging assays) of the fresh fruits of the biennial fruiting *Rubus idaeus* cultivars.

Cultivar	FC		FRAP		DPPH		ABTS		OH^•^ Scavenging Assay	
	mg/g (Caffeic Acid Equivalent)		μmol Fe(II)/g		mg TE/g		mg TE/g		mg TE/g	
	Min.–Max.	Mean ± SD	Min.–Max.	Mean ± SD	Min.–Max.	Mean ± SD	Min.–Max.	Mean ± SD	Min.–Max.	Mean ± SD
‘Glen Ample’	0.99–1.10	0.99 ± 0.01 ^a^	9.51–9.58	9.55 ± 0.04 ^a^	1.06–1.08	1.07 ± 0.01 ^a^	2.3–2.31	2.31 ± 0.01 ^a^	18.75–20.19	19.47 ± 0.72 ^a^
‘Laszka’	1.15–1.22	1.18 ± 0.03 ^b^	8.18–8.38	8.28 ± 0.01 ^b^	1.26–1.28	1.27 ± 0.01 ^b^	2.62–2.67	2.65 ± 0.03 ^b^	17.53–18.33	17.93 ± 0.42 ^b^
‘Radziejowa’	1.3–1.31	1.31 ± 0.01 ^c^	9.73–9.79	9.76 ± 0.03 ^c^	1.31–1.33	1.32 ± 0.11 ^c^	3.16–3.17	3.17 ± 0.01 ^c^	19.01–19.67	19.34 ± 0.33 ^a^

Note: Means for the content of phenolic compounds and antioxidant activity parameters followed by the same letter do not differ between the cultivars at a significance level of α = 0.05.

**Table 2:** Total content of phenolic compounds (FC) and antioxidant activity (FRAP, DPPH, ABTS, and OH^•^ scavenging assays) of juice from the fresh fruits of the biennial fruiting *Rubus idaeus* cultivars.

Cultivar	FC		FRAP		DPPH		ABTS		OH^•^ Scavenging Assay	
	mg/mL (Caffeic Acid Equivalent)		μmol Fe(II)/mL		mg TE/mL		mg TE/mL		mg TE/mL	
	Min.–Max.	Mean ± SD	Min.–Max.	Mean ± SD	Min.–Max.	Mean± SD	Min.–Max.	Mean ± SD	Min.–Max.	Mean ± SD
‘Glen Ample’	1.18–1.33	1.26 ± 0.08 ^b^	23.48–23.84	23.66 ± 0.18 ^a^	2.57–2.59	2.58 ± 0.01 ^b^	2.93–3.92	3.43 ± 0.49 ^a^	76.09–77.46	76.77 ± 0.68 ^a^
‘Laszka’	1.77–1.81	1.79 ± 0.02 ^a^	29.02–29.09	29.06 ± 0.04 ^b^	2.92–3.09	3.01 ± 0.08 ^a^	3.32–3.92	3.62 ± 0.30 ^a^	75.23–75.75	75.49 ± 0.26 ^b^
‘Radziejowa’	1.56–1.6	1.58 ± 0.02 ^a^	25.55–25.79	25.67 ± 0.12 ^c^	2.41–2,47	2.44 ±0.04 b^c^	2.91–3.41	3.16 ± 0.25 ^a^	71.97–72.66	72.315 ± 0.35 ^c^

Note: Means for the content of phenolic compounds and antioxidant activity parameters followed by the same letter do not differ between the cultivars at a significance level of α = 0.0.

## Data Availability

The data presented in this study are available in the article.
